# Determination of earthquake epicentres based upon invariant quantities of GRACE strain gravity tensors

**DOI:** 10.1038/s41598-020-64560-w

**Published:** 2020-05-06

**Authors:** Farzam Fatolazadeh, Kalifa Goïta, Rahim Javadi Azar

**Affiliations:** 1 CARTEL, Département de géomatique appliquée, Université de Sherbrooke, Québec, Canada; 2 Faculty of Geodesy and Geomatics Engineering, K. N. Toosi University of Technology, Tehran, Iran

**Keywords:** Seismology, Seismology, Geodynamics, Geodynamics

## Abstract

Investigation of regional and temporal variations in Earth’s gravitational field that are detected by the Gravity Recovery and Climate Experiment (GRACE) twin-satellites may be useful in earthquake epicentre determinations. This study focuses on monthly spherical harmonic coefficients that were extracted from GRACE observations, which were corrected for hydrological effects to determine earthquake epicentres. For the first time, we use the concept of deformation of Earth’s gravity field to estimate invariant components of strain tensors. Four different earthquakes (Iran, China, Turkey, Nepal) were analysed that occurred between 2003 and 2015 and under different hydrological regimes. Wavelet analysis was explored as a means of refining and reconstructing tectonic signals forming the disturbance gravitational potential tensor in the GRACE gravity field models. Dilatation and maximum shear were extracted from these tensors and used to map earthquake epicentre locations. Both components reached their maxima during months of the earthquakes (respectively, 11.78 and 4.93, Bam earthquake; 61.36 and 169.10, Sichuan-Gansu border earthquake; 2415.80 and 627.93, Elazig earthquake; 98.71 and 157.37, Banepa earthquake). For the aforementioned earthquakes, we estimated their respective epicentres in the ranges: *φ* = 29°–29.5° *λ* = 58.5°–59°; *φ* = 32.5°–33° *λ* = 105.5°–106°; *φ* = 38.5°–39° *λ* = 39.5°–40°; and *φ* = 27.5°–28° *λ* = 85°–85.5°. Overall, these results agree well with values from other sources. The advance that is provided by our method compared to other research is the ability of determining earthquake epicentres with magnitudes ≤7.5 based upon GRACE observations. However, the approach is of limited use for very deep earthquakes.

## Introduction

Earthquakes are natural geophysical processes that occur in certain locations across the surface of the globe. These seismic disturbances are found at the boundaries between tectonic plates (inter-plate) and in rift zones within plates (intra-plate). The rigid crust (whether continental or oceanic) and upper mantle constitute lithospheric or tectonic plates, which are cooler solid slabs of rock that ride on the hotter, more fluid deeper mantle. The surface tremors that characterise earthquakes result from gradual accumulation of strain and stress that is incurred when friction retards the movement of deeper rock strata from sliding against one another along a fault plane in opposing directions. When opposing fault faces are locked together, the continued movement of the surrounding lithosphere materials will continue to exert shear strain, i.e., energy that is stored, given that fractures can no longer extensively propagate along the fault to release the pressure. Given that the rocks cannot move freely, they deform (i.e., undergo dilatation^1^) and produce energy (like a sponge being squeezed), until they finally rupture. The accumulated energy that is released suddenly in the rupture is propagated as seismic waves. These waves spread outward through the surrounding crust from its initiation point or focus. When the waves reach the surface of the crust, they result in the shaking motions that we associate with an earthquake. The focal point at which the rupture occurs deep within the Earth is called the hypocentre, and the point on the Earth’s surface, which is located immediately above the hypocentre, is referred to as the epicentre of the earthquake.

Earthquake engineering is an ongoing and clearly important scientific endeavour in practical seismology. Scientists have used a variety of methods that can determine the time, location, and severity of an earthquake^2–4^. Traditional approaches for determining the epicentre of an earthquake, such as the triangulation method require at least three seismic data stations. This method uses the time difference between the earthquake and a monitoring station when each receives incoming P-waves and S-waves. The epicentre location then can be determined by drawing circles around each seismograph location based on these time differences, and locating the unique point where they intersect^5^.

A new era in earthquake science debuted with the launch of twin-satellites in 2002, i.e., GRACE (Gravity Recovery And Climate Experiment), which is based upon gravimetric measurements. In fact, an earthquake generates a redistribution of mass and, consequently, causes localised variation in Earth’s gravity field. GRACE satellites are capable of detecting these changes due to earthquakes. Sun and Okubo^6^ used dislocation theory for the first time to determine that on average, GRACE could detect an earthquake with a minimum magnitude of 7.5 on the Richter scale. Numerous studies following this idea investigated deformations in the form of surface displacements due to different earthquakes. Most combined GPS and GRACE data to observe co-seismic crustal vertical dilatation or subsidence^7–15^. Also, Chen *et al*.^16^ and Xu *et al*.^17^ further considered the effects of post-seismic deformation. All of these studies used analytical models or observations to understand tectonic deformation that was related to earthquakes.

In contrast to existing studies, the use of gravimetric data to aid the determination of an earthquake epicentre is a new idea. Hence, our motivation is to investigate its first-time implementation using GRACE observations. Two main questions are of interest: Is it possible to determine an earthquake epicentre using GRACE data? If yes, how do the magnitude and depth of the earthquake influence the process? Our objective is to propose an alternative method for locating lower magnitude earthquake epicentres based upon the deformational strain in gravity space that can be easily observed from satellite gravity data. Therefore, we propose a new and innovative approach for determining the invariant components of strain tensors of earthquakes using gravimetric observations.

We used the Global Land Data Assimilation System (GLDAS^18^) with an updated dataset (version 2.1) to remove hydrological effects from signals containing changes in mass distribution, thereby revealing tectonic signals in the remaining mass changes. First, GRACE data contamination from seasonal variations was reduced to improve tectonic signal extraction. We computed wavelet coefficients of the gravity signals from both hydrology and GRACE observations to filter and localise the desired signals based upon expansions of a Legendre polynomial in the frequency domain. The desired signals then were inserted into the gravity strain tensor to calculate dilatation and shear values. By considering two long periods before and after the earthquake, we determined the epicentre point of four different earthquakes. Last, the effect of earthquake depth on the estimated gravity strain components was considered. We added data from four other events, including two severe earthquakes (> 7 in magnitude) with great depth, to test the sensitivity of our model to the geometric depth parameter of the earthquakes.

## Case study tectonics

**Figure 1:**
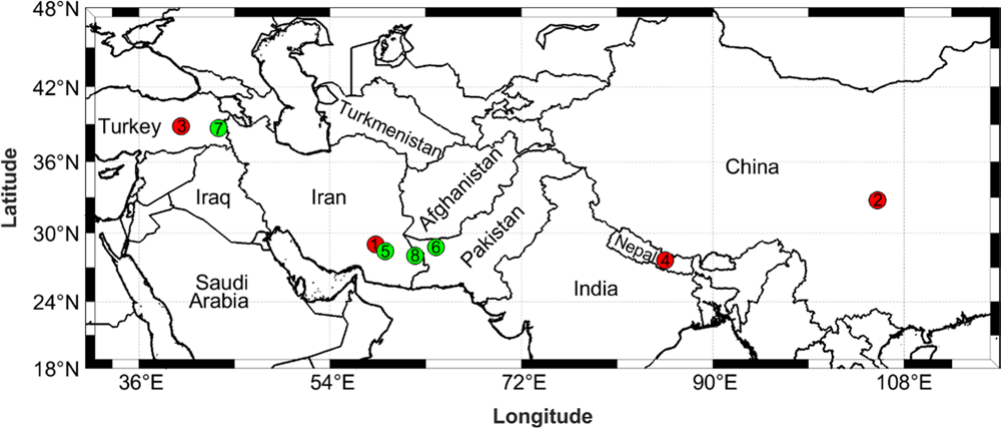
Locations of eight selected earthquakes. (1) Iran (Bam); (2) China (Sichuan-Gansu); (3) Turkey (Elazig); (4) Nepal (Banepa); (5) Iran (Khash); (6) Pakistan (Southwestern); (7) Turkey (Eastern); (8) Iran (Southeastern).

Eight different active seismic zones were considered in this study. The first four earthquakes are used to test the ability of the proposed approach to determine epicentres. Our criteria for choosing these areas included a long period of coverage, various hydrology disturbances, and seismic areas that were located on continents rather than oceans. Based upon these elements, we selected the Bam earthquake, which occurred in southeast Iran in 2003, the Sichuan-Gansu border earthquake that occurred in central China in 2008, the Elazig earthquake in eastern Turkey in 2010, and the Banepa earthquake in central Nepal near the Himalayas in 2015. Locations of these earthquakes are indicated by numbers 1 to 4 (red colour) in Fig. 1. We further describe some aspects of seismology that are related to these areas, including the general geological situation of the earthquake area, seismic records, tectonic conditions, and the earthquake mechanism (Table 1).

**Table 1:** Seismological properties of the case studies (USGS).

Name	Bam Earthquake	Sichuan-Gansu border earthquake	Elazig earthquake	Banepa earthquake
Country	Iran	China	Turkey	Nepal
Regional geology	Alluvial fans and argillaceous sandstones	Precambrian granitic rocks, sandstone and limestone	Anatolian Metamorphic Massifs and Basalt	Basement rock, gneiss, quartzite, phyllite and limestone
Time (UTC)	01:56:52	09:49:17	02:32:34	06:15:22
Date	26 December 2003	5 August 2008	8 March 2010	25 April 2015
Magnitude	6.6	6.2	6.1	6.1
Depth (USGS) (km)	8.5	6	12	10
Latitude, Longitude	28.995°N 58.311°E	32.756°N 105.494°E	38.864°N 39.986°E	27.628°N 85.540°E
Mercalli intensity scale	IX (Violent)	VII (Very Strong)	VIII (Severe)	VII (Very Strong)
Name of the Fault	The Bam Fault	The Longman Shan Fault	The East Anatolian fault	The Gorkha fault
Fault Plate Solution	Strike-slip	Thrust	Strike-slip	Strike-slip
Reasons for the earthquake	Combination of a small component of reverse-motion and strike-slip motion on the fault	As the Indian plate moves northward, it collides with Eurasian plate and continues moving forward into China	Displacement of left-lateral and right-lateral strike-slip on a northeast and northwest striking slip of the Anatolian fault	Collision of Indo-Australian and Asian plates

Four different major earthquakes that were characterised by great depth and magnitudes greater than 7, and which were located in Iran, Pakistan, and Turkey, were selected to better understand effects of earthquake depth on our proposed approach. Their locations are numbered 5 to 8 (green colour) in Fig. 1. Table 2 summarises the main characteristics and locations of the major earthquakes that were considered.

**Table 2:** Specifications of the selected great depth earthquakes (USGS).

Name	83 km East of Khash, Iran	Southwestern Pakistan	Eastern Turkey	Southeastern Iran
Date	16 April 2013	18 January 2011	23 October 2011	20 December 2010
Magnitude	7.7	7.2	7.1	6.7
Depth (USGS) (km)	80	68	18	12
Latitude, Longitude	28.033°N 61.996°E	28.777°N 63.951°E	38.721°N 43.508°E	28.412°N 59.180°E
Mercalli intensity scale	IX (Violent)	VII (Very Strong)	IX (Violent)	VIII (Severe)

## Methodology

One of the most important areas of research in geodesy is modelling of Earth’s deformation at local and global scales. Together with the study of causative factors that are involved in deformation and the provision of various computational methods for determining crustal displacements^19^. In deformation analysis, the Earth’s properties are usually compared under two situations: the reference or Lagrangian state (before deformation) and the current or Eulerian state (after deformation). The Earth-fixed three-dimensional Cartesian coordinate system of points on the ground is described as $\overrightarrow{R}=X,Y,Z$ in the Lagrangian state. In the Eulerian state, it is described as $\overrightarrow{r}=x,y,z$. The Cartesian coordinates of the displacement vector between these two states is denoted as $\overrightarrow{U}({U}_{i},{U}_{j},{U}_{k})=\overrightarrow{r}-\overrightarrow{R}+\overrightarrow{b}$, where $\overrightarrow{b}$ is the translation vector between the origins. In terms of displacement vectors, Lagrangian linear strain tensors (${e}_{yz}$) and Eulerian linear strain tensors (${e}_{yz}$) over the Earth’s crust would be expressed as follows^20^:

1 $${E}_{YZ}=\frac{1}{2}(\frac{\partial {U}_{j}}{\partial Z}+\frac{\partial {U}_{k}}{\partial Y}+\frac{\partial {U}_{i}}{\partial Y}\frac{\partial {U}_{i}}{\partial Z})$$

2 $${e}_{yz}=\frac{1}{2}(\frac{\partial {U}_{j}}{\partial z}+\frac{\partial {U}_{k}}{\partial y}+\frac{\partial {U}_{i}}{\partial y}\frac{\partial {U}_{i}}{\partial z})$$

In the literature of continuum mechanics, the theoretical concept of strain analysis is well known for its lack of dependence on the coordinate system. Grafarend and Voosoghi^21^ introduced this concept to Earth surface deformation analysis by exploring variations in the geometry of the surface through displacement fields that were computed from space mission measurements. This method is referred to as deformation of the gravity field of the Earth. A straightforward way of defining deformation in the gravitational field is to compare two different gravitational vector fields near a fixed point in space. Consider such a point, $\overrightarrow{R}=X,Y,Z$, and two different disturbing potential fields, *T* (before deformation) and *t* (after deformation), with the corresponding gravity disturbance vectors that are assigned to each: $\overrightarrow{\Gamma }=\frac{\partial T(\overrightarrow{R})}{\partial \overrightarrow{R}}$ and $\overrightarrow{{\rm{\gamma }}}=\frac{\partial t(\overrightarrow{R})}{\partial \overrightarrow{R}}$. Let these gravity disturbance vectors represent Euclidean coordinates in two corresponding gravity spaces. We used these coordinates to describe the strain in gravity space. The Lagrangian form of the gravity strain tensor^22^ is:

3 $$E(\overrightarrow{\Gamma })=\frac{1}{2}\left({\left(\frac{\partial \overrightarrow{{\rm{\gamma }}}}{\partial \overrightarrow{\Gamma }}\right)}^{T}\left(\frac{\partial \overrightarrow{{\rm{\gamma }}}}{\partial \overrightarrow{\Gamma }}\right)-I\right)$$

and the Eulerian form is:

4 $$e(\overrightarrow{{\rm{\gamma }}})=\frac{1}{2}\left(I-{\left(\frac{\partial \overrightarrow{\Gamma }}{\partial \overrightarrow{{\rm{\gamma }}}}\right)}^{T}\left(\frac{\partial \overrightarrow{\Gamma }}{\partial \overrightarrow{{\rm{\gamma }}}}\right)\right)$$

In this study, we used the Lagrangian strain formulation in numerical computations. These gravity strain tensors may be formulated using second-order space derivatives of the disturbing potential (gravity gradient disturbances). Let:

5 $$\frac{\partial }{\partial \overrightarrow{R}}{\left(\frac{\partial T}{\partial \overrightarrow{R}}\right)}^{T}=(\frac{\partial \overrightarrow{\Gamma }}{\partial \overrightarrow{R}})=B$$

6 $$\frac{\partial }{\partial \overrightarrow{R}}{\left(\frac{\partial t}{\partial \overrightarrow{R}}\right)}^{T}=(\frac{\partial \overrightarrow{{\rm{\gamma }}}}{\partial \overrightarrow{R}})=b$$

According to Eqs. 5 and 6 we have:

7 $$B=\left[\begin{array}{ccc}\frac{{\partial }^{2}T}{\partial {X}^{2}} & \frac{{\partial }^{2}T}{\partial X\partial Y} & \frac{{\partial }^{2}T}{\partial X\partial Z}\\ \frac{{\partial }^{2}T}{\partial Y\partial X} & \frac{{\partial }^{2}T}{\partial {Y}^{2}} & \frac{{\partial }^{2}T}{\partial Y\partial Z}\\ \frac{{\partial }^{2}T}{\partial Z\partial X} & \frac{{\partial }^{2}T}{\partial Z\partial Y} & \frac{{\partial }^{2}T}{\partial {Z}^{2}}\end{array}\right]$$

and

8 $$b=\left[\begin{array}{ccc}\frac{{\partial }^{2}t}{\partial {X}^{2}} & \frac{{\partial }^{2}t}{\partial X\partial Y} & \frac{{\partial }^{2}t}{\partial X\partial Z}\\ \frac{{\partial }^{2}t}{\partial Y\partial X} & \frac{{\partial }^{2}t}{\partial {Y}^{2}} & \frac{{\partial }^{2}t}{\partial Y\partial Z}\\ \frac{{\partial }^{2}t}{\partial Z\partial X} & \frac{{\partial }^{2}t}{\partial Z\partial Y} & \frac{{\partial }^{2}t}{\partial {Z}^{2}}\end{array}\right]$$

The GRACE satellite is able to recover the gravity changes in terms of spherical harmonic coefficients. Indeed, spherical coefficients are the solution to the partial differential equation of the disturbance potential that satisfies the Laplace equation^23^.

9 $$T(r,\varphi ,\lambda )=\frac{GM}{a}\mathop{\sum }\limits_{n=2}^{N}{\left(\frac{a}{r}\right)}^{n+1}\mathop{\sum }\limits_{m=0}^{n}\mathop{\sum }\limits_{\alpha =0}^{1}\Delta {\bar{C}}_{nm\alpha }{\bar{Y}}_{nm\alpha }(\varphi ,\lambda )$$

10 $${\bar{Y}}_{nm\alpha }(\varphi ,\lambda )={\bar{P}}_{nm}(\sin \,\varphi )\{\begin{array}{c}\cos \,m\lambda \,\alpha =0\\ \sin \,m\lambda \,\alpha =1\end{array}\Delta {\bar{C}}_{nm\alpha }=\{\begin{array}{c}\Delta {\bar{C}}_{nm}\,\alpha =0\\ \Delta {\bar{S}}_{nm}\,\alpha =1\end{array}$$

with spherical coordinates $(r,\varphi ,\lambda )$, and fully normalised geopotential spherical harmonic coefficients $\Delta {\bar{C}}_{nm\alpha }$. We fixed the radial coordinate at $r=a$, where $a=6371000\,{\rm{m}}$ is the mean Earth radius. We used monthly geophysical coefficients of GRACE release-5 that were processed by the Center for Space Research (CSR05, University of Texas Austin), which are produced on the maximum degree and order 60. These are publicly available. As is the case in other studies, the GRACE coefficient with degree 2 and order 0 with its large errors was replaced by the coefficient that was obtained from Satellite Ranging Laser (SLR)^24^. These coefficients also contain mass variation due to seismic and hydrological effects^25^. Therefore, the removal of hydrological effects is an integral part of the process to obtain refined signals that are related to the earthquake. Hydrological variables were derived from the Noah Land Surface Model process level-4^26^, which was obtained from GLDAS-2.1 model simulations for the study period (temporal resolution: 1 month, spatial resolution: (1^°^). The simulations contained 36 land surface fields from 2000 to present^18,27^. They include the most effective hydrological variables, such as surface runoff, subsurface runoff, soil moisture content from surface to 2 m depth, canopy water content, and surface snow amount. No data for groundwater were provided in the GLDAS model. Yet, we assumed the effects of this component are negligible. The correction process consisted of converting the hydrological signals from their space (water layer thickness) to gravity space. This conversion is performed by applying Love numbers^28^. All of these were transformed, in turn, to coefficients through harmonic analysis, to maintain consistency with GRACE observations. The corresponding coefficients were subtracted from GRACE coefficients to obtain data that were corrected for hydrological effects:

11 $$\Delta {\bar{C}}_{nm\alpha }^{Corrected}=\Delta {\bar{C}}_{nm\alpha }-\Delta {\bar{C}}_{nm\alpha }^{Hydrology}$$

Unfortunately, GRACE has limited precision in determining locations due to its low spatial resolution. Panet *et al*.^29,30^ introduced localised wavelet analysis to reconstruct regional spatiotemporal potential changes in the form of GRACE coefficients. In comparison with Fourier transformations, wavelet functions decompose the main signal to extract its regional behaviour at different frequencies by transfers in time and expansion or compression (scale change) in amplitude. Spherical wavelets are a special type of wavelets that form radial base functions over the sphere by increasing the signal-to-noise ratio over the region. In this regard, their scaling properties are described by a scaling function. In this study, a non-orthogonal cubic polynomial was considered as a scale function (${\hat{\phi }}_{J}$), together with its wavelet (${\hat{\Psi }}_{J}$), based upon the following equations:

12 $${\hat{\phi }}_{J}(n)=\{\begin{array}{lc}{(1-{2}^{-J}n)}^{2}(1+{2}^{-J+1}n) & for\,n\in [0,{2}^{J}]\\ 0 & for\,n\in [{2}^{J},\infty ]\end{array}$$

13 $${\hat{\Psi }}_{J}(n)=\sqrt{{({\hat{\phi }}_{J+1}(n))}^{2}-{({\hat{\phi }}_{J}(n))}^{2}}$$

These wavelets were inserted directly into Eq. (9) to reconstruct the disturbing potential (T).

14 $$T(r,\varphi ,\lambda )=\frac{GM}{a}\mathop{\sum }\limits_{n=2}^{N}\mathop{\sum }\limits_{m=0}^{n}\mathop{\sum }\limits_{\alpha =0}^{1}{\hat{\Psi }}_{J}(n)\Delta {\bar{C}}_{nm\alpha }^{Corrected}{\bar{Y}}_{nm\alpha }(\varphi ,\lambda )$$

Considering Lagrangian formulation, we defined the following scenarios: i) Before the earthquake, each target month would be considered the current disturbing potential (t), whilst the mean of the two years prior to the earthquake would be considered as the reference disturbing potential (T). ii) After the earthquake, each target month would be considered the current state disturbing potential (t), whilst the mean of the two years following the earthquake would be considered as the reference disturbing potential (T).

Then, with $b=\left(\frac{\partial \overrightarrow{{\rm{\gamma }}}}{\partial \overrightarrow{R}}\right)=\left(\frac{\partial \overrightarrow{{\rm{\gamma }}}}{\partial \overrightarrow{\Gamma }}\right)\left(\frac{\partial \overrightarrow{\Gamma }}{\partial \overrightarrow{R}}\right)=\left(\frac{\partial \overrightarrow{{\rm{\gamma }}}}{\partial \overrightarrow{\Gamma }}\right)B$, the disturbance gravity strain tensors that are referred to Cartesian coordinates then become:

15 $$E(\overrightarrow{\Gamma })=\frac{1}{2}({B}^{-1}{b}^{2}{B}^{-1}-I)$$

16 $$e(\overrightarrow{{\rm{\gamma }}})=\frac{1}{2}(I-{b}^{-1}{B}^{2}{b}^{-1})$$

Since the strain is a symmetric tensor, only six components are required to specify it at a given point^31^:

17 $$E=[\begin{array}{ccc}{\varepsilon }_{11} & {\varepsilon }_{12} & {\varepsilon }_{13}\\ {\varepsilon }_{21} & {\varepsilon }_{22} & {\varepsilon }_{23}\\ {\varepsilon }_{31} & {\varepsilon }_{32} & {\varepsilon }_{33}\end{array}]$$

In general, ${\varepsilon }_{11}$, ${\varepsilon }_{22}$, and ${\varepsilon }_{33}$ represent the components of elongation in the three coordinate directions. The off-diagonal components of the strain tensor are related to angular deformation (or shear) of pairs of lines that are initially oriented in three coordinate directions. The cubical dilatation is the trace of the strain tensor and represents the body volume change that is incurred during deformation. According to the Lagrange strain tensor, dilatation (*D*) and shear (*SH*) are respectively computed such that:

18 $$D(\varphi ,\lambda )=\mathop{\sum }\limits_{i=1}^{3}{\varepsilon }_{ii}(\varphi ,\lambda )={g}_{max}+{g}_{min}$$

19 $$SH(\varphi ,\lambda )=\sqrt{Trace{(\varepsilon (\varphi ,\lambda ))}^{2}-4{\rm{\det }}(\varepsilon (\varphi ,\lambda ))}={g}_{max}-{g}_{min}$$

where ${g}_{max}$ and ${g}_{min}$ are the maximum and minimum eigenvalues of strain gravity tensor. Dilatation components display isotropic features, which are not dependent upon direction. However, the maximum shear is non-isotropic and, therefore, geometrical interpretation of the maximum value of shear is a key factor in earthquake epicentre determination.

To determine an earthquake’s epicentre using GRACE data, we introduced the following method. The average two years prior to the year of the earthquake are considered as the current state, whilst the average of the two years after the year of the earthquake can be considered as the reference state. The decision to use two years may be explained by signal processing concepts. As previously mentioned, dilatation and shear can be computed from the gravity gradient changes that are extracted from GRACE data. Taking the second derivative of the data, however, weakens the signal^32^. To analyse mass change details that are induced by an earthquake, we need to consider a long period of time. Therefore, we assumed that considering changes over two years would be reasonable to acquire these details. By constructing a gravity strain tensor from GRACE observations, as explained above, the maximum shear appears at the earthquake’s epicentre. Generally, the best results are achieved when the reference year is considered as a year in which a large mass change (usually in the form of earthquakes) has not occurred in and around the study area. We tested our method on four different seismic areas with various hydrological disturbances to provide a more comprehensive analysis surrounding the computation of the location of the earthquake’s epicentre.

To investigate uncertainties in epicentre results, we proposed a method for estimating the contributions of errors that are induced by the GRACE observations in the computation of dilatation and maximum shear. More precisely, we defined upper/lower and right/left bounds of the errors for the maximum shear values. In each case, the interval size between the bounds depends upon the location of the maximum shear in the earthquake’s region, and its changes in surrounding areas. In order to define these bounds, we examined the changes in maximum shear using different window sizes. In all cases, a size of 1° was found to be sufficient. Outside this 1° range, changes in maximum shear appear to be either negligible or nonexistent. To illustrate the process, let us consider the case of the Bam earthquake. According to the position of the epicentre, two profiles of maximum shear were computed in the longitudinal direction. The first one was for the lower bound situated at 28.5°N, while the second was for the upper bound at 29.5°N. The difference between the maximum shear values of the two profiles indicates the uncertainty in the north-south direction. The same process was used to compute the profiles in the latitudinal direction. The profiles were computed for the left and right bounds, located respectively at 58°E and 59°E. The difference that was found between their maximum values was considered as the uncertainty in the maximum shear in the east-west direction.

In practice, in mathematical geodesy, a region will be defined in terms of the uncertainties, which normally is an error ellipse. This region is a confidence area that is based upon maximum errors according to coordinate axes. This assumption does not hold in this study, since the maximum shear is an invariant parameter of the strain tensor, and is not dependent on a coordinate system. Under these circumstances, defining an error ellipse does not make sense. The solution is to use an alternative approach that produces similar uncertainties in all directions.

In this regard, Hoover^33^ introduced an algorithm for the true determination of positioning and navigation. He determined the probability of having the true position inside a confidence circle. We used this idea here to calculate the radius *r*_*cc*_ of that confidence circle, the centre of which corresponds to the estimated epicentre position for a given earthquake, and radius to maximum error of the shear in all directions. Let us consider respectively a_sh_ and b_sh_ as the errors on maximum shear values in the east-west and north-south directions, as explained above. The ratio *C*_*sh*_ between these errors can be computed from the following equation:

20 $${C}_{sh}=\frac{{b}_{sh}}{{a}_{sh}}$$

As the margin of errors increases, the diameter of the error circle will increase. To ensure that this circle would cover the entire error region, we used a 95% confidence level by defining a scale coefficient (*K*) in Eq. 21, which is then used to compute the radius *r*_*cc*_ of the confidence circle (Eq. 22):

21 $$K=1.960790+0.004071\,{C}_{sh}+0.114276\,{{C}_{sh}}^{2}+0.371625\,{{C}_{sh}}^{3}$$

22 $${r}_{cc}=K.{a}_{sh}$$

## Results and Discussion

**Figure 2:**
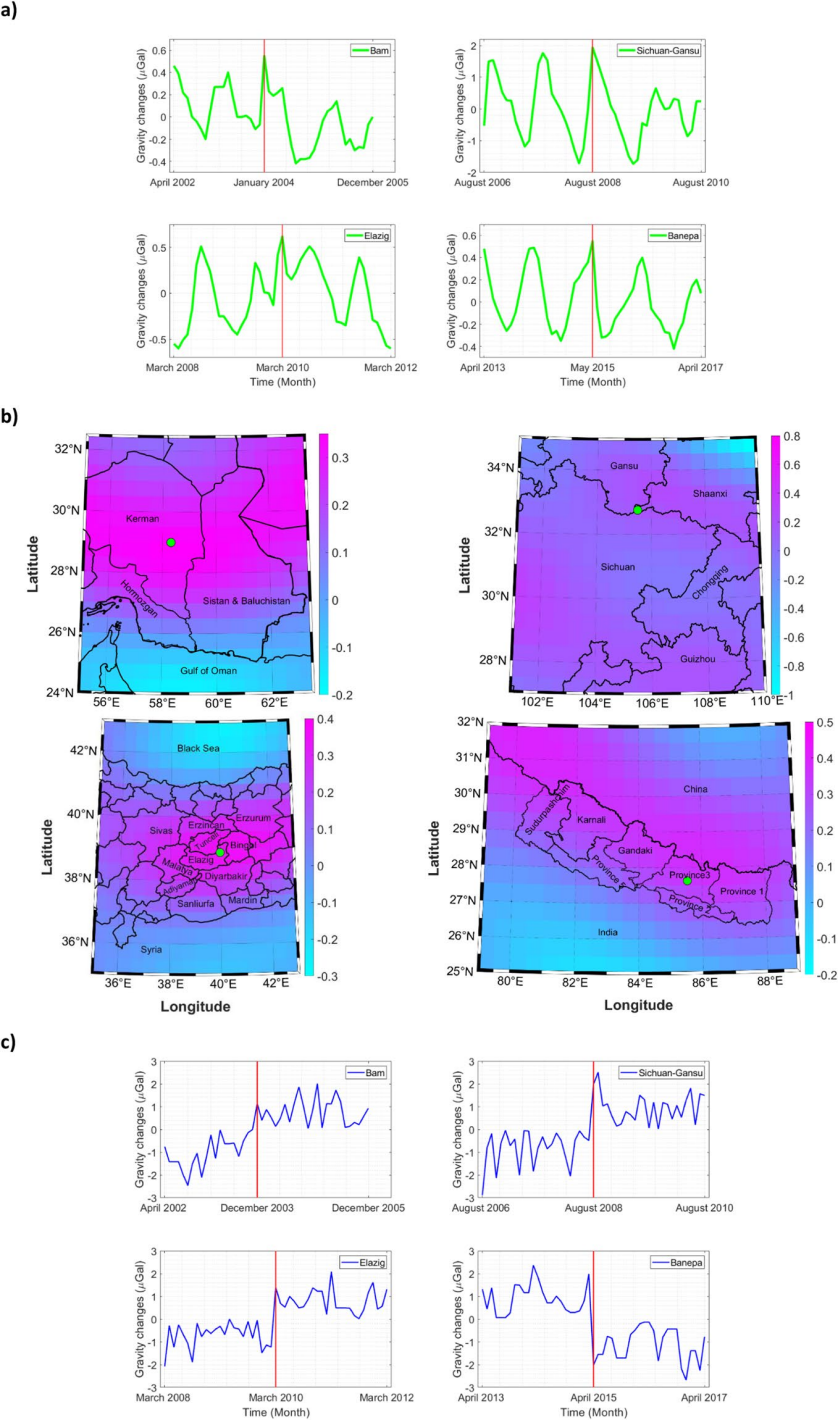
Computed mean gravity changes for the different sites: (**a**) Gravity changes due to hydrological effects; (**b**) Spatial variations of gravity changes due to hydrological effects in the month of the earthquake; (**c**) Gravity changes after the corrections of the hydrological effects.

As mentioned in the previous section, removal of hydrological signals from GRACE observations is necessary to retrieve tectonic signals. Following the methodology that was previously explained (Section 3), as a first step, we computed long-term mean gravity changes due to the hydrological effects for a period of about 4 years over the study regions (i.e. 2 years before the earthquake occurrence and 2 years after). Differences between each month and a mean of the whole period (4 years) were considered to compute these anomalies. The time series of these gravity variations for each earthquake are shown in Fig. 2a in green color. As expected, they display seasonal patterns due to the cyclic nature of hydrological features (e.g. precipitations). The maximum values of the changes are reached in the month of earthquake (which is materialised by the vertical red line in time series of Fig. 2). Because Bam and Banepa earthquakes happened in the last days of the month, the maximum changes occurred the following month.

In a second step, we mapped the gravity variations due to hydrological effects in the specific month of each earthquake. Figure 2b shows the pattern of these variations over the four different seismic areas that were considered. The maximum gravitational changes apparently occurred near the tectonic areas. As shown on Fig. 2b, a positive gravitational change occurred in the vicinity of all the earthquakes, showing significant anomalies in land surface states. These may be explained by high precipitation or other components of the water budget in each area during the earthquake month. Table 3 shows the maximum gravity variations in each case. The values vary from 0.51 to 1.94 µGal (1 Galileo = 1 cm/s^2^). This highest value was found for the Chinese earthquake. From the literature, the maximum gravitational changes that have been recorded for the most severe earthquake in the 21^st^ C. are about 10 µGal for both the Sumatra earthquake in 2004 (M 9.2) in Indonesia, and the Tohoku-Oki earthquake 2011 in Japan (M 9.0)^34,35^. Other severe earthquakes, such as Maule earthquake 2010 (M 8.8) in Chile, recorded a gravity change of about 5 µGal^36^. Accordingly, the values of gravitational changes that were introduced by hydrological effects, which were computed in this study for the four earthquakes considered (Table 3), appear non-negligible, particularly in the case of China. If applied to the case of Chile, such values would introduce errors varying from 10 to 20% of the total gravitational changes. Therefore, removing the effects of hydrological contributions from the monthly mass variations that were measured by GRACE is required to improve earthquake signal retrieval.

**Table 3:** Maximum gravity variations due to hydrological effects over the regions that were affected by earthquakes.

	Bam earthquake	Sichuan-Gansu border earthquake	Elazig earthquake	Banepa earthquake
Maximum gravity variations (µGal)	0.55	1.94	0.62	0.51

In the third step, we have corrected the hydrological effects on all the entire time series considered in the study (not only for the months of the earthquakes). Gravity variations were computed after these corrections. The results are shown in Fig. 2c for each earthquake. As it can be seen on this figure, the correction of hydrological effects has almost removed the seasonal pattern in the mass variations signals. The jump of the gravity values related to the occurrence of the earthquake can be easily observed in each case in Fig. 2c. Sichuan-Gansu border earthquake exhibits the maximum variations as expected (about 4 µGal). Positive and negative changes in Fig. 2c translate the expansion and contraction in the two sides of the fault plane of each earthquake, confirming the seismic signals.

**Figure 3:**
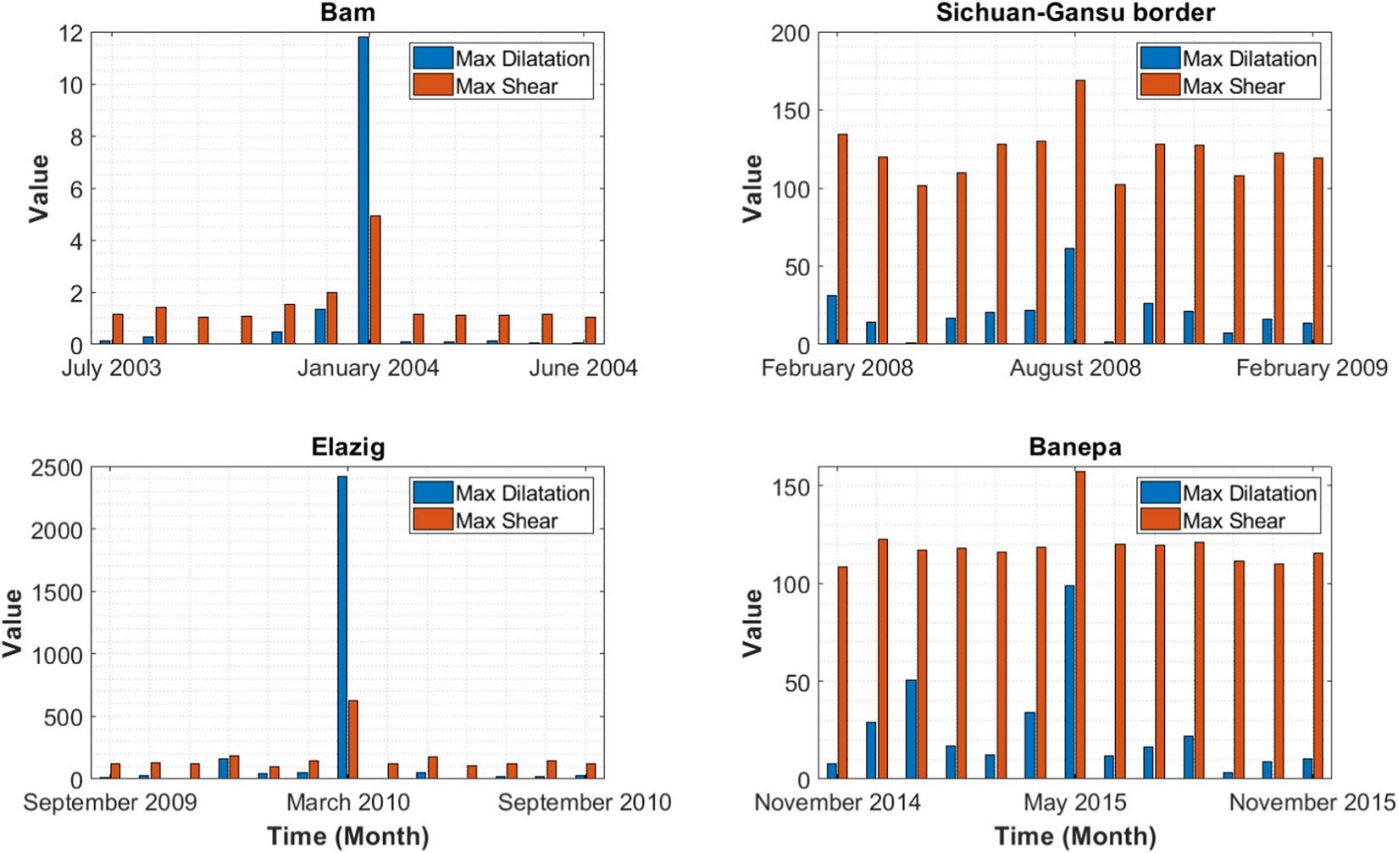
Time series of invariant components of strain gravity tensors over each earthquake location.

Using the monthly gravity variations computed after the hydrological effects corrections (Fig. 2c), the invariant components of the strain gravity tensor were calculated in the periods that were considered (two years prior to and two years after the main earthquake). The method that was used for the computations is detailed in Section 3. Figure 3 indicates the maximum values of dilatation and shear for the four earthquakes. The calculated dilatation and shear are averaged over the region under consideration. We have shown only a period of six months before and after the earthquake (Fig. 3), because calculated values of the dilatation and shear were very low outside this window. The timeframe for each event is as follows: Bam earthquake, July 2003 to June 2004 (since there is no GRACE observation data for June 2003); Sichuan-Gansu border earthquake, February 2008 to February 2009; Elazig earthquake, September 2009 to September 2010; and Banepa earthquake, November 2014 to November 2015.

As shown in the time series for each earthquake in Fig. 3, the values of dilatation and shear (no measurement unit) reach their maxima at the earthquake occurrence time. For the Bam earthquake, the amount of dilatation and shear before the earthquake is insignificant. However, as the date of the earthquake approached, the values started to change, and increased steadily in the two months prior to the earthquake. Since the Bam earthquake occurred in late December 2003, maximum values appeared in January 2004, due to the monthly availability of GRACE data. After the earthquake, the dilatation and shear dropped dramatically toward their original states. The same behaviour could be observed for the Elazig earthquake. The behaviours that were observed seem logical in relation to the Earth’s physical properties. They can be explained by the viscoelasticity of tectonic plates, which return to their original states, after being transformed into the inner layers of the Earth^37^. As no earthquake event occurred between February and June 2004 in the Bam area, dilatation and shear remained constant, as shown in Fig. 3. The time series of the Elazig earthquake shows the same trend as the Bam earthquake. Yet, values of dilatation and maximum shear are not significant for the periods outside the earthquake occurrence month. This could be explained by the fact that during the two-year period considered, only the earthquake of 8 March 2010 occurred in the Elazig locale and vicinity. Therefore, dilatation and shear remained negligible before and after the earthquake.

Although the maximum value of dilatation and shear occurred in the month of earthquake for the other two earthquakes, the process is not linear. This behaviour can be affected by the occurrence of small earthquake events. For the Sichuan-Gansu border and Banepa earthquakes, Fig. 3 also shows an increase in gravity tensor components as the events approached, with decreases thereafter. This response is particularly true with respect to the dilatation component. When compared to the other two earthquakes, the values of the gravity tensor components remained much higher and significant during the whole year for both Banepa and Sichuan-Gansu, with some small exceptions. In fact, 62 lower magnitude earthquakes occurred near the China site during the time frame under consideration, while 33 events were recorded around the Nepal site^38^. Both areas were very active during the time period studied here, and this is well reflected in the GRACE-based approach that we proposed. Yet, Fig. 3 did not clearly show the severe earthquake (magnitude 7.9, depth 20 km), which occurred near the Sichuan-Gansu border on 15 May 2008. This suggests a possible influence of earthquake depth on the gravity strain components. This issue will be addressed in the subsequent section with different cases.

**Figure 4:**
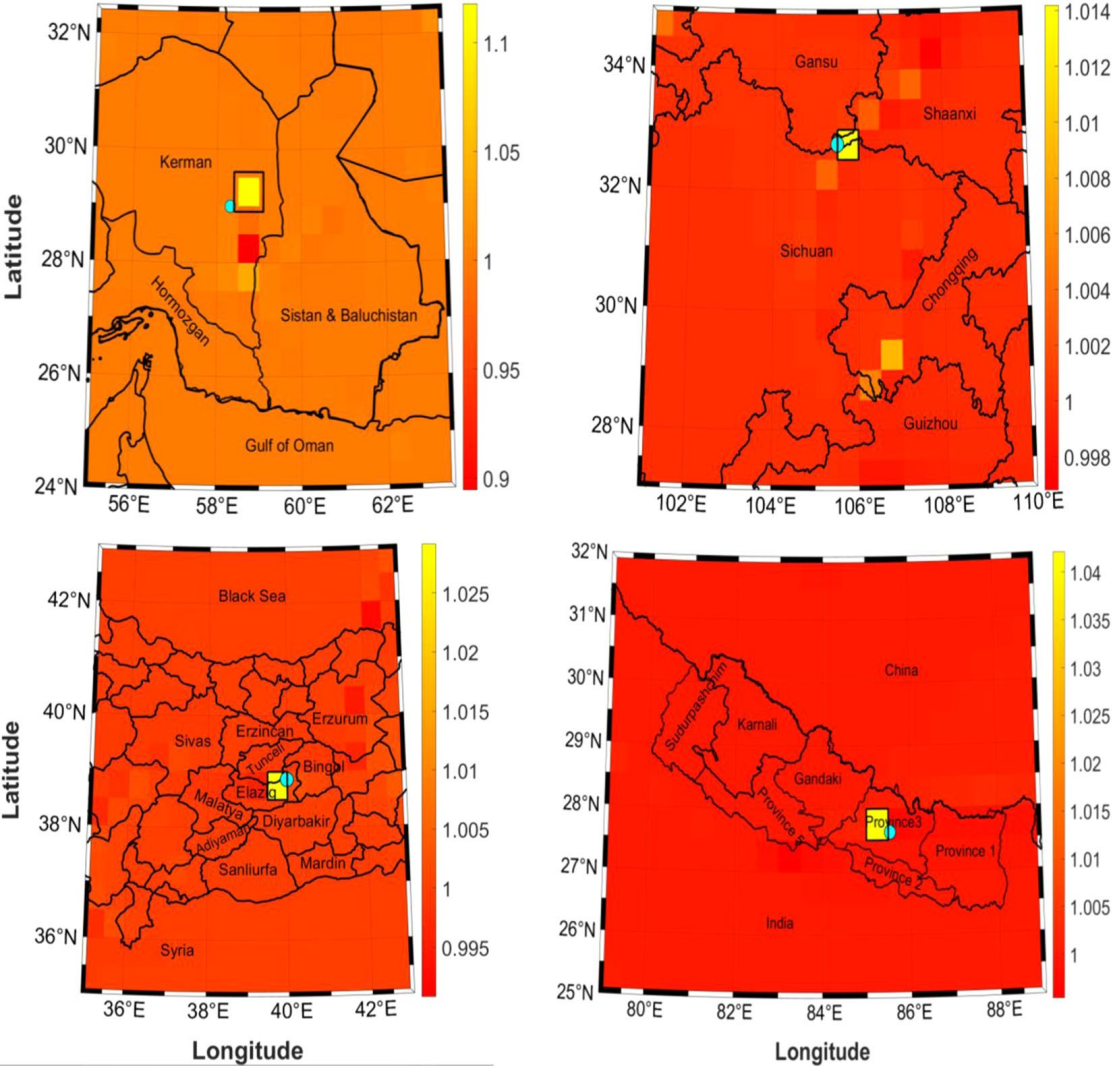
Epicentre determined for each earthquake based upon maximum shear (yellow square). Cyan circle shows the epicentre that was extracted by UGGS. First row from left to right: Bam, Sichuan-Gansu border. Second row from left to right: Elazig, and Banepa.

As dilatation and shear reach their maximum values at the earthquake occurrence time, determining the epicentre could be possible. Therefore, we applied our method, as explained in Section 3. Accordingly, we considered an average two years before and after the earthquake year as the reference and current states, respectively, to form gravity strain tensors and compute maximum shear and dilatation for each earthquake. Figure 4 shows each earthquake epicentre location obtained using the approach. As both the maximum shear and dilatation produced the same result, only those that were extracted using maximum shear are presented. The cyan circle indicates the epicentre location that was provided by USGS. The black square outline surrounding the yellow-colored pixel (pixel with maximum shear) includes the location of the epicentre determined by our method.

It must be noted that large deformations caused by earthquakes are typically highly localised, with associated signals residing in high frequency bands^39^. Unfortunately, due to the altitude of GRACE, retrieval of reliable gravitational changes is only feasible in the low-to-medium frequency bands, because of an aliasing problem with higher wavelengths^40^. Therefore, applications of spaceborne gravimetry to earthquake studies are seriously limited by the satellite’s current spatial resolution (1^°^ by 1^°^). To smooth the decay speed of the spectrum in the gravitational and gradient changes that were observed by GRACE, together with the deformation that was associated with the earthquake, we implemented wavelet analysis to localise our data and improve the resolution (see Section 3). In the localisation method, we assumed that the preferred location of the epicentre of the earthquake corresponds to the centre of the main cell that has been identified with the maximum shear value. Yet, the epicentre could actually reside anywhere inside the cell. As a result, the method is not able to determine a definite position for the epicentre. Alternatively, the result is given in terms of ranges of latitude and longitude comprising the point. In general, Fig. 4 clearly shows that the locations of maximum peaks in the invariant components of the strain gravity tensor are very close to the earthquake epicentre, as determined by USGS.

With a little greater focus on Fig. 4, it can be noted that several cells of different colours are present, especially for the Bam area, which may be explained by the fault kinematic concept in continuum mechanics. The different coloured cells that are related to the Bam earthquake show changes in slip rate^41^. This pattern yields the direction of the dominant fault line in the region, which is in south-north direction in this case. Moreover, this rate change can be also seen in the Chinese earthquake area, showing that the Sichuan fault line is oriented in a southwest to northwest direction.

The comparison between the locations that were determined in this study and the epicentre coordinates that were provided by three different sources is presented in Table 4. The three sources are the USGS, the International Seismological Centre-Global Instrumental Earthquake Catalogue (ISC-GEM), and the International Institute of Earthquake Engineering and Seismology (IIEES). Promising agreement is generally found.

**Table 4:** Comparison between the locations of earthquake epicentres according to from our method and other sources.

	Bam Earthquake	Sichuan-Gansu border earthquake	Elazig earthquake	Banepa earthquake
USGS	$\varphi ={28}^{^\circ }59{\prime} 42{\prime\prime}$ $\lambda ={58}^{^\circ }18{\prime} 39\,.\,6{\prime\prime}$	$\varphi ={32}^{^\circ }45{\prime} 21.\,6{\prime\prime}$ $\lambda ={105}^{^\circ }29{\prime} 38.\,4{\prime\prime}$	$\varphi ={38}^{^\circ }51{\prime} 50.04{\prime\prime}$ $\lambda ={39}^{^\circ }59{\prime} 9.\,6{\prime\prime}$	$\varphi ={27}^{^\circ }37{\prime} 40.8{\prime\prime}$ $\lambda ={85}^{^\circ }32{\prime} 24{\prime\prime}$
ISC-GEM	$\varphi ={28}^{^\circ }51{\prime} 00{\prime\prime}$ $\lambda ={58}^{^\circ }15{\prime} 00{\prime\prime}$	$\varphi ={32}^{^\circ }44{\prime} 06{\prime\prime}$ $\lambda ={105}^{^\circ }30{\prime} 50.\,4{\prime\prime}$	$\varphi ={38}^{^\circ }47{\prime} 24{\prime\prime}$ $\lambda ={40}^{^\circ }01{\prime} 48{\prime\prime}$	$\varphi ={27}^{^\circ }40{\prime} 23.88{\prime\prime}$ $\lambda ={85}^{^\circ }41{\prime} 16.08{\prime\prime}$
IIEES	$\varphi ={29}^{^\circ }00{\prime} 36{\prime\prime}$ $\lambda ={58}^{^\circ }15{\prime} 36{\prime\prime}$	$\varphi ={32}^{^\circ }48{\prime} 00{\prime\prime}$ $\lambda ={105}^{^\circ }28{\prime} 48{\prime\prime}$	$\varphi ={38}^{^\circ }52{\prime} 12{\prime\prime}$ $\lambda ={39}^{^\circ }59{\prime} 24{\prime\prime}$	$\varphi ={27}^{^\circ }36{\prime} 36{\prime\prime}$ $\lambda ={85}^{^\circ }50{\prime} 06{\prime\prime}$
Our approach	*φ* = 29°–29.5° *λ* = 58.5°–59°	*φ* = 32.5°–33° *λ* = 105.5°–106°	*φ* = 38.5°–39° *λ* = 39.5°–40°	*φ* = 27.5°–28° *λ* = 85°–85.5°

We used the only available authoritative sources (USGS, ISC-GEM and IIEES) to validate our computed results for the epicentre of each earthquake. As mentioned above, the spatial resolution of the GRACE dataset makes it impossible to accurately determine the location of the epicentre^8^. Therefore, the most reasonable method for validating the results is to consider ranges of differences in terms of distance. The approximate differences between our method for computing the longitude and latitude of each earthquake epicentre and other sources are displayed in Table 5. As shown, these differences are smaller than the dimensions of a GRACE cell in both the latitude and longitude directions, indicating that the approach performs well. The differences in longitude direction appear quite higher than those found in the latitude direction. Further investigations will be needed in forthcoming studies to improve the results in the longitude direction. The spatial resolution of GRACE data remains one of the major limitations of the proposed method. The use of optimisation methods such as a genetic algorithm or neural network with real-time seismic data from other sources could eventually lead to better spatial resolution for GRACE through downscaling, and contribute to resolve the major limitation of the approach proposed.

**Table 5:** Differences in epicentres computed by our method and other sources.

	Data Source	Distance in latitude and longitude, respectively(km)
Bam earthquake	USGS	18-87
	ISC-GEM	29-103
	IIEES	23-90
Sichuan-Gansu border earthquake	USGS	28-54
	ISC-GEM	26-54
	IIEES	33–53
Elazig earthquake	USGS	15–58
	ISC-GEM	23–56
	IIEES	14–59
Banepa earthquake	USGS	42–55
	ISC-GEM	41–70
	IIEES	54–83

## Error analysis

**Figure 5:**
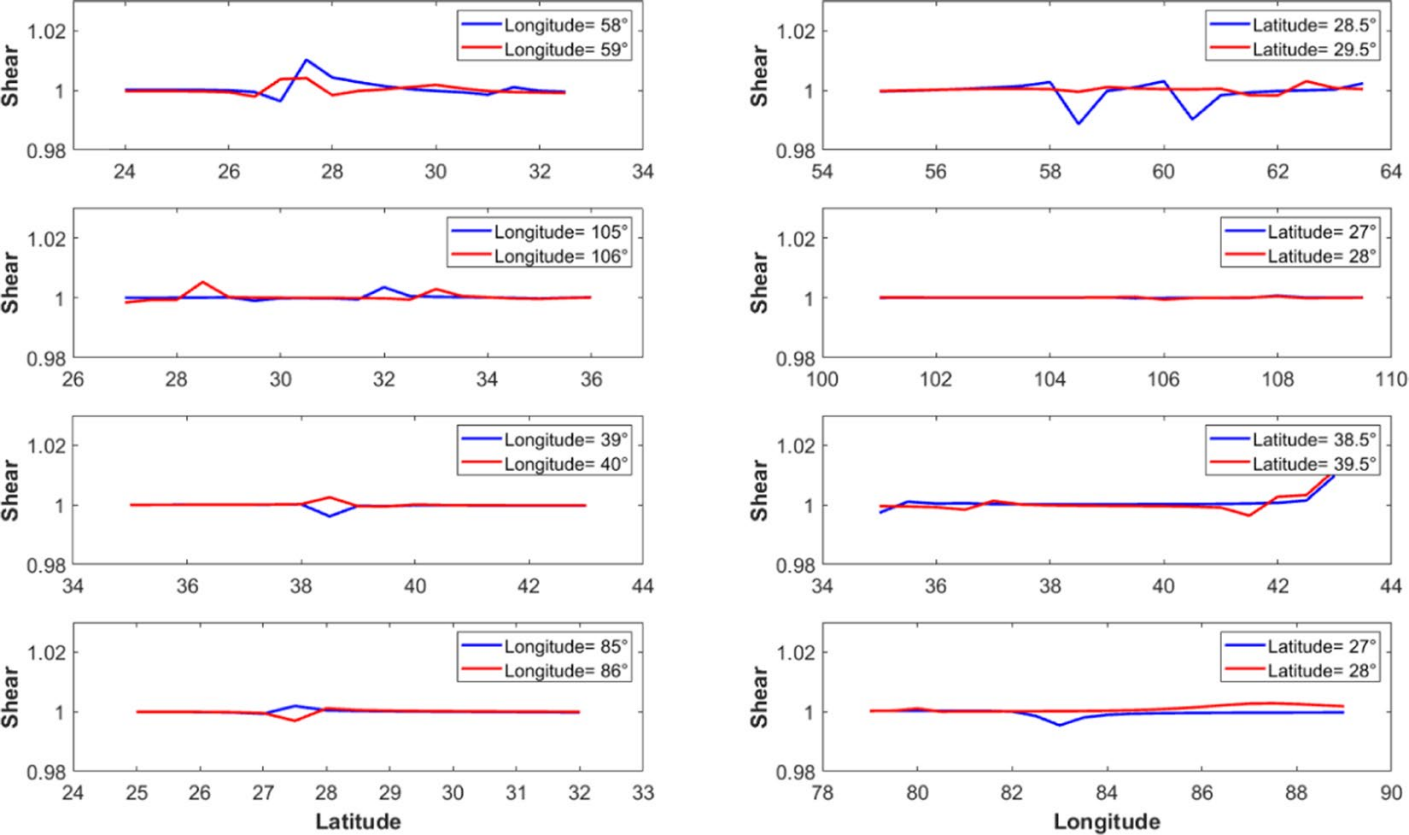
Maximum shear profiles in latitude (left column) and longitude (right column) directions. First to fourth row: Bam, Sichuan-Gansu, Elazig and Banepa.

As explained in Section 3, the profiles of maximum shear were computed using bounds located at 0.5° apart from the epicentre, in order to estimate the uncertainties both in north-south and east-west directions. Figure 5 shows the variations of the maximum shear in latitude and longitude directions. The changes appear quite small in general.

Table 6 shows the corresponding uncertainties computed. Since the shear is a non-unit invariant parameter, the values of the uncertainties are unitless. The maximum errors were observed in latitude direction for all the examined earthquakes, except for Sichuan-Gansu in China, which display the higher errors on the longitude axis. Overall, the errors found are very small on both axes (less than 1.5%). The resulting computed confidence circle radius are also indicated in Table 6, in terms of maximum errors on the shear parameter. The values are very weak for Sichuan-Gansu and Elazig earthquakes, and remain less than 3% in any case. This indicates that the proposed approach is robust and performs well.

**Table 6:** Uncertainties computed for shear component over epicentre (no unit).

	Bam region	Sichuan-Gansu border region	Elazig region	Banepa region
Error (longitude profile) Error (% of total shear)	0.0077 0.60%	0.0063 0.62%	0.0064 0.62%	0.0018 0.17%
Error (latitude profile) Error (% of total shear)	0.0144 1.28%	0.0034 0.33%	0.0143 1.38%	0.0074 0.71%
Radius of Circle error Error (% of total shear)	0.0296 2.64%	0.0161 1.58%	0.0289 2.80%	0.0146 1.40%

### Effect of earthquake depth

**Figure 6:**
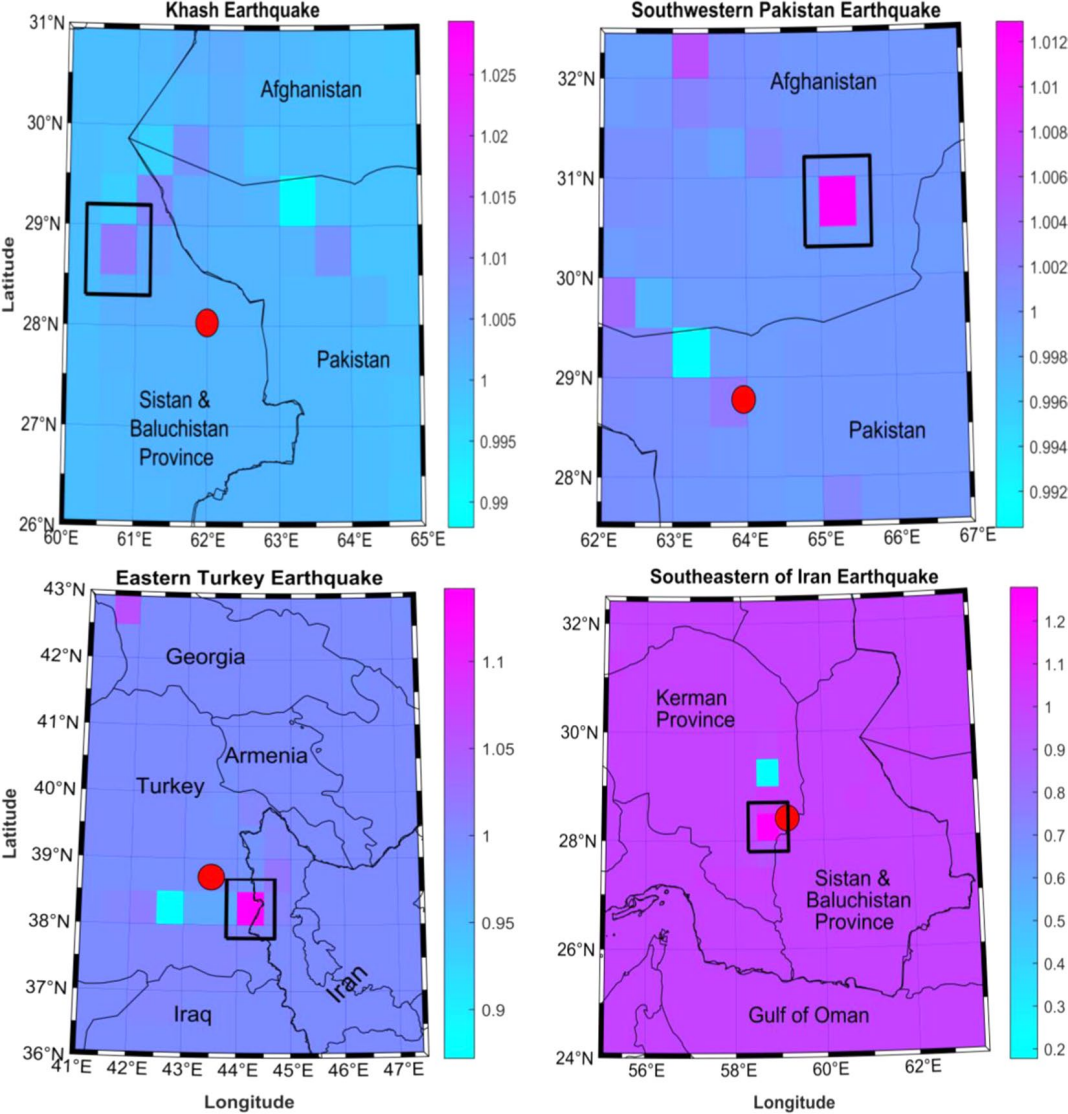
Location of epicentre obtained (black square) compared with the main earthquake indicated in a red circle. The top row shows deep earthquakes (left: Khash, right: Southwestern Pakistan), and the bottom row shows shallow earthquakes (left: Eastern Turkey, right: for Southeastern Iran). The scale indicates a range between maximum (epicenter location) and minimum shear.

Sensitivity analysis of gravitational changes as a function of fault parameters is a well known process in earthquake mechanism^42^. In this study, we wanted to understand whether the earthquake depth could affect the epicentre location determined by the proposed approach. As indicated in Section 3, we applied the method to four different earthquakes cases with depth varying from 12 to 80 km (See Table 2). The results are compiled in Fig. 6. It can be clearly seen that the estimated epicentre positions lack accuracy for deep earthquakes. In fact, despite their higher magnitudes (greater than 7), the epicentres determined respectively for the Khash (80 km depth) and Southwestern Pakistan (68 km depth) earthquakes are far from their real positions. In both cases the distance of separation is near 200 km. However, the epicentre for shallow earthquakes (less than 15 km in this study) could be determined with reasonable accuracy, according to GRACE spatial resolution. This includes the initial four earthquakes considered, whose depths were between 6 and 12 km (Table 1), plus Southern Iran (12 km depth, Table 2). For Eastern Turkey (depth 18 km), the distance to the real epicentre position is about 80 km. This is far better than the distance found for deep earthquakes (depth >60 km), but less accurate than the results found for more shallow events with depth less than 15 km. Overall, it could be concluded from the analysis that the GRACE-based approach is sensitive to earthquake depth. It appears better for shallow depth events (less than 15 km), occurring in a magnitude range of 5 to 7. This is consistent with a previous study of Fatolazadeh *et al*.^43^ who reported that increasing earthquake depth generates weakened gravity changes. It also explains why the GRACE-based approach did not capture clearly the severe earthquake of magnitude 7.9 and depth of 20 km, which occurred near the Sichuan-Gansu border in 15^th^ May 2008 in China (Fig. 3).

## Conclusion

Our approach brings new findings in the use of invariant components of gravity strain tensor, including dilatation and shear, extracted from GRACE satellite gravimetric observations to determine earthquakes epicenters location. The approach is based on the second derivatives of potential changes as gravitational gradient changes of monthly geophysical coefficients of GRACE data along with the GLDAS model and localised wavelet analysis. The results showed that the values of dilatation and shear increase by approaching the earthquake time and reach their maximum at the occurrence of the earthquake. Due to GRACE data structure and spatial resolution, epicenter location can only be expressed in terms of range of latitudes and longitudes. Hence, for the four different earthquakes considered to demonstrate the applicability of the approach, epicenter was estimated in the range of *φ* = 29°–29.5°, *λ* = 58.5°–59° for the Bam earthquake (Iran), *φ* = 32.5°–33°, *λ* = 105.5°–106° for Sichuan-Gansu border earthquake (China), *φ* = 38.5°–39°, *λ* = 39.5°–40° for Elazig earthquake (Turkey), and *φ* = 27.5°–28°, *λ* = 85°–85.5° for Banepa earthquake (Nepal). All the results are in good agreement with authoritative referenced sources, with distance differences less than the spatial resolution of GRACE; indicating thus the potential of the approach. The correction of hydrological effects from GRACE data is important for a better computation of tectonic signals in the approach presented. The resulting gravity strain tensor components showed a clear linkage with earthquake event. Even small magnitude earthquakes (5 to 6) manifest themselves with slight variations in maximum dilatation and shear values. Therefore, according to the cases tested, GRACE seems able to detect earthquakes with a magnitude of less than 7.5. However, most accurate results were found for only shallow depth earthquakes (less than 15 km). The effects of depths and other geometric parameters of the earthquake should be further addressed in forthcoming studies to better define the domain of applicability of the method. The coarse spatial resolution of GRACE data constitutes a limitation for accurately determining epicenter location, as it falls somewhere inside the identified cell with maximum shear value. Downscaling approaches may eventually improve GRACE spatial resolution and help to have a better location of earthquakes epicenters using the approach.

## Data Availability

Spherical harmonic coefficients of GRACE are available at ftp://podaac-ftp.jpl.nasa.gov/allData/grace/L2/CSR/RL05/. Information on the seismic event mentioned in the paper can be found in the USGS earthquake catalog from https://earthquake.usgs.gov/earthquakes/search/. Hydrological parameters of the GLDAS model version 2.1 can be downloaded from https://hydro1.gesdisc.eosdis.nasa.gov/data/GLDAS/GLDAS_NOAH10_M.2.1/. Moreover, all Matlab codes used in this study are accessible from the first author (corresponding author) upon scientific request.
